# Prevalence of orthokeratinized odontogenic cyst: A retrospective clinicopathological study

**DOI:** 10.6026/973206300221518

**Published:** 2026-03-31

**Authors:** Gurmej Singh Warval, Jasbir Kaur, Rohit Chopra, Natasha Attri, Ujjwal Chawla, Yogita Garg

**Affiliations:** 1 Department of Oral and Maxillofacial Surgery, Government Dental College, Patiala, India; 2 Department of Dentistry, Guru Gobind Singh Medical College and Hospital, Faridkot, India; 3 Department of Periodontics, Guru Gobind Singh Medical College and Hospital, Faridkot, India; 4 Department of Periodontics, Government Dental College, Amritsar, Punjab, India; 5 Department of Oral and maxillofacial surgery, Dasmesh Institute of Research and Dental Science, Faridkot, India; 6 Department of Periodontics, Government Dental College, Patiala, India

**Keywords:** Orthokeratinized odontogenic cyst (OOC), prevalence, odontogenic cysts, mandible, clinicopathological features, impacted tooth, histopathology, unilocular radiolucency, retrospective study, jaw cystic lesions

## Abstract

Orthokeratinized odontogenic cyst is a distinct developmental jaw cyst commonly misinterpreted at the clinical and radiographic level due to overlap with other keratinizing and tooth-associated cysts. Hence, 1,200 biopsy specimens were analyzed, identifying 30 histologically confirmed OOC cases. OOC constituted 10.0% of all odontogenic cysts and 2.5% of the total biopsy cohort. The majority of lesions affected males between 21-30 years and were predominantly located in the posterior mandible, frequently involving impacted teeth, especially the mandibular third molar. The study highlights its rarity, limited tissue aggression and the importance of microscopic confirmation for accurate diagnosis and appropriate treatment planning.

## Background:

Orthokeratinized odontogenic cyst (OOC), formerly grouped under keratocystic lesions, is now recognized as a distinct developmental odontogenic cyst with unique biological behaviour [1]. OOC differs from keratocystic odontogenic tumor (KCOT) in histopathology, showing orthokeratinized epithelial lining and granular cell layer without PTCH gene mutations commonly seen in KCOT [2]. Unlike KCOT, OOC demonstrates a low recurrence rate, less aggressive growth and absence of syndromes' association, indicating a more favourable prognosis [3]. Most OOCs occur in the mandible, particularly the posterior region and commonly affect young to middle-aged males [4]. Radiographically, OOC commonly presents as a well-defined unilocular lesion, often asymptomatic and associated with impacted teeth, especially the third molar [5]. Due to its clinical similarity to other odontogenic cysts, especially KCOT, OOC remains frequently misdiagnosed, leading to inconsistent prevalence reporting across studies [6]. Accurate prevalence assessment requires combined clinical, radiologic andhistopathological correlation to improve diagnostic precision and treatment planning [7]. Chronic odontogenic infections can lead to progressive bone involvement when diagnosis is delayed, emphasizing the role of early clinical and radiographic evaluation in preventing complications such as osteomyelitis and deep-seated maxillofacial infections [8]. Histopathological evaluation is essential for the accurate diagnosis of orthokeratinized odontogenic cyst, as its clinical and radiographic features often overlap with other odontogenic cystic lesions [9]. Orthokeratinized odontogenic cyst is a relatively uncommon developmental odontogenic cyst with distinct clinicopathological characteristics [10]. Therefore, it is of interest to study Orthokeratinized odontogenic cyst to determine its prevalence and biological behaviour.

## Methodology:

This retrospective, cross-sectional clinicopathological study was conducted. All biopsy records of jaw cystic lesions diagnosed in past 2 years were retrieved from archival registers and the institutional pathology database. During the study period, a total of 1,200 oral and maxillofacial biopsy specimens were received, of which 300 were confirmed odontogenic cysts. After histopathological slide verification, 30 cases met the diagnostic criteria for Orthokeratinized Odontogenic Cyst (OOC) and were included for analysis. Cases with incomplete records, recurrent cysts lacking original diagnostic slides, poorly preserved tissue or lesions showing Para keratinization or tumor-based histologic features suggestive of KCOT were excluded. Diagnosis was confirmed by re-evaluation of Hematoxylin and eosin stained sections by experienced oral and maxillofacial pathologists to ensure consistency with known OOC morphology, defined by an orthokeratinized stratified squamous epithelial lining, a prominent granular cell layer and the absence of Para keratinization. Clinical data including age, sex and lesion location, presenting symptoms and radiographic findings from available imaging were extracted from patient files. Prevalence was calculated as the proportion of confirmed OOC cases relative to the total number of odontogenic cyst diagnoses and the total biopsy cohort. Data were tabulated and summarized using descriptive statistics, presenting frequencies and percentages for categorical variables and mean values for age and cyst size. Associations between OOC occurrence and demographic or clinical variables were evaluated using standard categorical comparison tests based on data distribution. All patient information was anonymized prior to analysis and the study followed institutional ethical policies for retrospective archival research.

## Results:

A total of 1,200 oral and maxillofacial biopsy specimens and 300 histologically confirmed odontogenic cysts were reviewed during the study period. Thirty (30) cases were verified as Orthokeratinized Odontogenic Cyst (OOC/OOC-also referred to as OOC catalogued as OOC), forming the final prevalence sample.The highest number of OOC cases (10 patients, 33.3%) were observed in the 21-30 year age group, while no cases were recorded in children aged 0-10 years (Table 1 - see PDF). Most cysts were of moderate dimension, with 16 lesions (53.3%) measuring 2-4 cm in diameter. The mean cyst size was 4.2 cm, indicating that while OOC is less aggressive than tumor-classified keratinizing cysts, it can still reach clinically significant size before detection (Table 2 - see PDF). A clear relationship with teeth was noted in most cases. Impacted tooth association was present in 22 patients (73.3%), while tooth displacement was documented in 8 cases (26.7%) and root resorption in only 2 cases (6.7%), confirming that destructive effects on adjacent structures are uncommon (Table 3 - see PDF). On slide re-evaluation, almost all OOCs displayed the expected keratinization profile, showing a uniform orthokeratinized epithelial surface in all 30 cases (100%), a granular cell layer in 29 cases (96.7%) and complete absence of par keratinization or satellite/daughter cysts, supporting the developmental rather than neoplastic nature of the lesion (Table 4 - see PDF). Clinical impression before biopsy corresponded with the histologic diagnosis in only 60% of cases, reflecting frequent similarity in presentation to KCOT and dentigerous cysts. The remaining lesions were initially suspected as KCOT (26.7%), dentigerous cyst (10.0%) or other cyst types (3.3%), showing that diagnostic confusion persists when pre-biopsy evaluation relies on radiography and symptoms alone (Table 5 - see PDF). Based on institutional archives, the prevalence of OOC was 10.0% among all odontogenic cysts and 2.5% of the total biopsy cohort, a proportion consistent with its recognition as an uncommon but clinically important cystic lesion. Demographically, OOC affected males more frequently (20 males, 66.7%) than females (10 females, 33.3%). The mandible was the dominant site (25 cases, 83.3%), especially the posterior mandibular region (21 cases, 70.0%) and the most frequent tooth involvement was the mandibular third molar (18 cases, 60.0%) (Table 6 - see PDF).

## Discussion:

Histopathological assessment remains critical for differentiating odontogenic cysts and tumors with overlapping microscopic features, ensuring accurate diagnosis and classification [11]. Early clinicopathological studies emphasized the structural and epithelial variations among odontogenic cysts, forming the basis for their current classification [12]. Mosquea-Taylor *et al.* highlighted the importance of correlating clinical, radiographic and histological findings in odontogenic lesions to avoid diagnostic errors [13]. Distinct histopathological patterns have been described as essential criteria for identifying specific odontogenic cyst subtypes [14]. Radiographic characteristics play a complementary role in the diagnosis of odontogenic cysts and help assess lesion extent and behavior [15]. Lou and Li demonstrated that epithelial differentiation and keratinization patterns are key indicators of biological behavior in odontogenic lesions [16]. Recent institutional studies have contributed updated prevalence data and reinforced the clinicopathological relevance of retrospective analyses [17]. Case reports have illustrated uncommon presentations of odontogenic cysts, emphasizing the need for careful diagnostic evaluation [18]. Santosh reviewed the biological behavior and diagnostic challenges associated with odontogenic cysts and tumors, stressing the role of histopathology [19]. Clinical studies have shown that early diagnosis and appropriate management of odontogenic cysts can reduce recurrence and complications [20]. Buser emphasized the significance of bone biology and healing response in maxillofacial lesions, relevant to surgical management of cystic pathologies [21]. Population-based studies have provided insight into the frequency and distribution of odontogenic cysts in different demographic groups [22]. Reports from otolaryngology literature have highlighted the potential for odontogenic cysts to involve adjacent anatomical regions, complicating diagnosis [23]. Recent pathology-based studies reaffirmed the importance of standardized histopathological criteria in distinguishing odontogenic cyst variants [24]. Kahraman *et al.* reported clinicopathological features and outcomes of odontogenic cysts, supporting the value of retrospective prevalence studies [25].

## Conclusion:

Orthokeratinized odontogenic cyst is an uncommon developmental odontogenic cyst characterized by indolent and non-aggressive biological behaviour. It demonstrates a strong predilection for young adult males with frequent posterior mandibular and impacted third-molar association. Routine histological confirmation is critical to avoid misdiagnosis and prevent unnecessary aggressive management.
